# Editorial: Thought leaders in robotics and AI

**DOI:** 10.3389/frobt.2023.1265092

**Published:** 2023-08-08

**Authors:** Herbert G. Tanner

**Affiliations:** Center for Autonomous and Robotic Systems, University of Delaware, Newark, DE, United States

**Keywords:** evolutionary robotics, field robotics (FR), cyber physical system (CPS), datasets and data quality, learning and adaptation

The purpose of this short Research Topic is to highlight some directions within the long-term research vision of selected robotics experts. The intention is to spark discussion and debated on these subjects as a means of promoting the importance of the associated technical problems and moving the research further forward. The three papers that comprise this Research Topic are titled, in alphabetical order of their authors: “*From the lab to the field with evolutionary field robotics*,” by Howard; “*Progress and challenges in adaptive robotics*,” by Nolfi; and “*Towards understanding and synthesis of contact-rich anthropomorphic motions throught interactive cyber-physical human*” [platfrom] by Yoshida.

The paper by Howard draws connections between work in the area of field robotics, and research related to evolutionary robotics. The thesis of the paper is that the two research fields are in fact closely related, with some of the stronger links being developed around their common focus on the (robot’s) environment. Whereas evolutionary robotics is focused on environmentally-adapted robots that perform effectively in complex environments and offer us insights into the mechanisms of natural evolution, field robotics is expected to utilize unstructured natural environments to test, validate, and demonstrate meaningful robotic function. The paper coins the new term of “evolutionary field robotics” to describe activities in the direct intersection of these two research areas. This particular subarea identified is primarily developing and exploiting evolutionary mechanisms to provide solutions to field robotic problems, by leveraging the aforementioned intellectual connections between two seemingly different existing fields of study.

The paper by Nolfi is conceptually related to Howard’s paper in the sense that it discusses adaptive robotics—which is not to be confused with adaptive control of robots. Adaptive robotics is defined here as the Research Topic of methodologies that allow robots to develop new skills autonomously through the utilization of evolutionary and/or learning algorithms. In this light, the connection to evolutionary robotics that Howard is discussing is obvious; one possible difference in the treatment of the two authors is that Nolfi highlights perhaps a bit more the role of (deep) learning methods. Nolfi’s paper thus focuses on the state of the art in adaptive robotics, emphasizing on work that aims at autonomous skill evolution through minimal human intervention, and the utilization of model-free methods. This paper directs the spotlight on a few current research challenges in this area, promoting solutions to these problems as key enablers for future technology: knowledge re-use and environment model acquisition. The paper also draws some links to optimization, system identification, dynamical systems, and their role in the adaptation process. What is probably more striking, however, is the recurring appearance of the importance of the process by which the robot interacts with its environment, in the context of end-to-end learning.

The article by Yoshida, on the other hand, is perhaps a bit more focused methodologically and application-wise. This paper paints in some broad strokes a vision for how highly interactive cyber-physical humanoid platforms can be exploited for the synthesis of contact-rich, natural, whole-body anthropomorphic motions. Yoshida points out the fact that data sets for whole-body contact and interaction forces are currently missing, and this is a bottleneck for the development and realization of similar behaviors in robots. In this sense, the availability of an experimental platform capable of producing such datasets could be a game changer in the area. Along this line of work, Yoshida claims that the combination of physical humanoid robots with digital twins, and the generation and annotation of human motion databases that could support continuous learning, can be key for advancing the area forward. To bring and integrate these components together, Yoshida envisions a bridge between a model-based analysis framework and a data-driven methodology, an example of which could be based on inverse optimal control. The paper identifies several technical challenges that lay ahead on the road for achieving this breakthrough, and candidate targets for future research. As such, Yoshida identifies the challenge of combinatorial complexity associated with multi-contact planning, the unavailability of contact-rich human motion databases, and the absence of an appropriate cyber-physical system that can be utilized as a measurement instrument for the acquisition of such data.

One interesting common thread through these three papers is the emphasis they place on several aspects in which the environment and the context in which robot morphology and function is developed, becomes critically important. Each of these papers highlights this observation in different ways: Howard raises the issue in the context of evolution; Nolfi makes this point for adaptation; Yoshida stresses the argument within a discussion on deliberate planning and control synthesis. For instance, the point that Howard makes about the detrimental effect of highly abstracted and oversimplified simulated environments, echoes in Yoshida’s argumentation on the importance of having high-fidelity digital human twins, and Nolfi’s advocacy for processes for model acquisition as well as knowledge representation. From this high-level viewpoint, one could argue that enriched, both simulated as well as physical robot environments, are especially important for the evolution and development of future robot technology. They are a key ingredient in the effort to transition our robots out of our the controlled and sterile space of our research labs and into the complex, messy and unpredictable real world, where they need to deploy in order to decisively prove their worth and serve us all better.

